# A prospective study of comparing waist circumference and BMI as predictors for the kidney damage progression

**DOI:** 10.1371/journal.pone.0321012

**Published:** 2025-04-29

**Authors:** Jou-Yin Chen, Yukiko Wagatsuma

**Affiliations:** 1 Department of Clinical Trials and Clinical Epidemiology, Graduate School of Comprehensive Human Sciences, University of Tsukuba, Tsukuba, Ibaraki, Japan; 2 Department of Clinical Trials and Clinical Epidemiology, Faculty of Medicine, University of Tsukuba, Tsukuba, Ibaraki, Japan; Shahjalal University of Science and Technology, BANGLADESH

## Abstract

**Objective:**

Chronic kidney disease (CKD) is irreversible and linked with various adverse health outcomes and diminished quality of life. Although obesity is recognized as a risk factor for the progression of kidney damage, reliance solely on body mass index (BMI) to measure obesity has been increasingly questioned. The use of other indicators that reflect more on abdominal adiposity like waist circumference (WC) have been proposed. This study aims to determine whether WC can serve as an alternative predictor of kidney damage progression.

**Methods:**

This prospective study enrolled individuals with normal kidney function during their annual health checkups from April 2016 to March 2019. Data on BMI, WC, WC-related devices, health-related lifestyle, and comorbidities were collected at baseline. WC was categorized using various definitions and analyzed for its association with the risk of kidney damage progression, taking into account BMI categories. The participants were monitored until March 2023 to observe kidney damage progression.

**Results:**

Out of the 4,129 participants, WC showed a higher risk of kidney damage progression in males (HR=1.01–1.39, p-value<0.05). These associations were not observed in females. After adjusting for BMI categories, the associations disappeared. Males in the overweight BMI category, defined as a BMI ≥ 25 kg/m^2^, showed a significantly increased risk of kidney damage progression (HR = 1.69, p-value < 0.0001).

**Conclusions:**

The findings indicate that waist circumference significantly affects the progression of kidney damage in males. However, the study also reaffirms BMI as a dependable predictor of kidney damage. It underscores the importance of maintaining normal ranges for both BMI and waist circumference to reduce the risk of progressing kidney damage.

## Introduction

Chronic kidney disease (CKD) affects over 10% of the global population and involves the gradual progression of kidney damage. CKD is a significant contributor to global mortality and has been identified as such in numerous studies [1]. The global burden of CKD has consistently increased over the past 27 years [2]. Although the loss of kidney function is usually irreversible and progressive, early detection and preventive interventions can slow the progression of kidney damage and reduce mortality among patients with CKD [3].

Obesity is recognized as one of the major risk factors for CKD. Previous studies have underscored the risk of developing CKD associated with higher body mass index (BMI) levels [4,5]. However, recent years have seen growing skepticism about the efficacy of BMI as a sole measure of obesity. The American Medical Association has issued a statement indicating that BMI is an inappropriate measure of body health across different ethnic groups, genders, and age ranges due to its limitations [6]. Furthermore, the relationship between BMI and individuals’ metabolic profiles may not always be consistent. A previous study demonstrated that compared to Western populations, Asians of similar age and BMI tend to have higher abdominal adipose tissue [7]. This suggests that relying solely on BMI to define adiposity status is insufficient.

To better characterize adiposity, waist circumference (WC) serves as an indicator of abdominal obesity and is strongly correlated with visceral adipose tissue, which is linked to metabolic risks [8]. Several indices have been developed to assess abdominal obesity. These include the waist-to-height ratio (WHtR), which adjusts for height and is calculated by dividing WC by height [9]; the Waist-BMI ratio, obtained by dividing WC by BMI [10]; the Body Roundness Index (BRI), which considers body shape and eccentricity to describe fat distribution using WC and height [11]; the Weight-Adjusted Waist Index (WWI), which incorporates WC and weight [12]. Other indices include the Conicity Index (CI), and the A Body Shape Index (ABSI), both of them utilize measurements of weight, height, and WC to evaluate abdominal obesity [13,14]. This measurement offers insights into the distribution of central adipose tissue and accommodates variations in body sizes. Given recent doubts about the effectiveness of BMI in defining obesity, establishing WC as a reliable indicator for predicting CKD is becoming increasingly important. Previous studies have shown an increased risk of CKD associated with elevated WC. However, these studies have mostly been cross-sectional [15]. Moreover, although several abdominal obesity indices have been widely developed, the relationship between these indices and CKD progression remains underexplored, limited by the lack of longitudinal studies, or insufficiently understood in healthy populations [16–18]. Thus, comprehensive prospective studies are needed to confirm these findings. This prospective study aims to confirm whether WC is a predictive indicator of kidney damage progression and assess the predictability of WC-related indices in relation to the risk of kidney damage progression.

## Materials and methods

### Study subjects

The study subjects were participants of annual health checkups at a regional hospital in Ibaraki, Japan. Recruitment began in April 2016, with approximately 5,000 community residents and workers attending the health check-up annually. During the health checkups, various measures were taken, including demographic and anthropometric variables, blood and urine tests, and responses to a health-related lifestyle questionnaire. Participants with an eGFR ≥ 60 and no proteinuria at baseline were included in the study. Exclusion criteria included participants with missing values for WC or eGFR at baseline, those who did not attend follow-up visits, or those with incomplete eGFR data during the follow-up period.

### Study design

This prospective cohort study enrolled subjects with normal kidney function from April 1, 2016 to March 31, 2019. The date on which each subject first enrolled was established as the baseline point. Demographic data, anthropometric variables, comorbidities, and blood test results were collected at baseline. The study monitored the progression of kidney damage until March 31, 2023.

### Definitions and measurements

WC was measured to one decimal point in centimeters by trained hospital staff. Classification into high and low WC groups followed the criteria set by the World Health Organization (WHO) and the Japan Society for the Study of Obesity (JASSO). According to WHO standards, a WC of more than 90 cm for males and 80 cm for females was considered high [19]. Additionally, JASSO recommended a high WC classification for males with a WC exceeding 85 cm and females over 90 cm [20]. WC-related indices including the WHtR, Waist-BMI ratio, BRI, CI, ABSI, and WWI. These abdominal obesity indices were calculated by the following formula [10,16,18,21–23]:


$$WHtR=\frac{WC(cm)}{height(cm)}$$

$$Waist-BMIratio=\frac{WC(cm)}{BMI(kg/m^{2})}$$

$$BRI=364.2-\frac{365\times \sqrt{{\left(1-(WC(cm)/2\pi \right)}^{2}}}{{\left(0.5\times height(cm)\right)}^{2}}$$

$$Conicityindex=\frac{WC(m)}{0.109\times \sqrt{weight(kg)/height(m)}}$$

$$ABSI=\frac{WC(m)}{{BMI}^{\frac{2}{3}}\times {height(m)}^{\frac{1}{2}}}.$$

$$WWI=\frac{WC(cm)}{\sqrt{weight(kg)}}$$

WHtR, CI, and ABSI were categorized into high and low groups based on cutoff points reported in previous studies. The cutoff points were 0.52 for males and 0.49 for females for WHtR [21]; 1.275 for males and 1.285 for females for CI [18]; and 0.0822 for males and 0.0795 for females for ABSI [24].

BMI was calculated as weight (kg) divided by height (m) squared. A high BMI group was defined as BMI ≥ 25 kg/m^2^ for males and BMI ≥ 23 kg/m^2^ for females, reflecting the generally slimmer body size of Japanese women, in line with recommendations for the Asian population [25].

The estimated glomerular filtration rate (eGFR) was determined using a formula specifically tailored for the Japanese population: eGFR = 194 × serum creatinine ^(−1.094)^ × age ^(−0.287)^ × 0.739 (if female) [26]. Progression of kidney damage was characterized by an eGFR falling below 60 mL/min/1.73m^2^ or the occurrence of proteinuria, tested using the urine protein dipstick (Uropaper α III ®, Eiken Chemical Inc., Tokyo, Japan) and defined as urine protein exceeding 30 mg/dL [27,28].

Health-related lifestyle factors and comorbidities such as hypertension, diabetes, and dyslipidemia were included as covariables. Hypertension was defined as systolic blood pressure over 140 mmHg or diastolic blood pressure over 90 mmHg. Diabetes was defined by a fasting glucose level exceeding 126 mg/dL, hemoglobin A1c (HbA1c) over 6.5%, or current use of anti-diabetic medication. Dyslipidemia was defined by any of the following criteria: triglycerides ≥ 150 mg/dL, high-density lipoprotein < 40 mg/dL, or low-density lipoprotein ≥ 140 mg/dL [29]. Health-related lifestyles were assessed through a self-reported questionnaire, which is a standard form mandated by the Ministry of Health in Japan. Key lifestyle factors included exercise (engaging in more than 30 minutes of exercise at least twice per week for over a year), smoking status (smoking within the last month) and alcohol consumption (drinking alcohol (such as sake, shochu, beer, whisky etc.)), which were selected as covariates for this study.

### Statistical analysis

Baseline characteristics of the study subjects were presented as numbers (percentages) for categorical variables and means (standard deviations) for continuous variables. We compared the characteristics of males and females, as well as participants with and without progression of kidney damage, using the chi-square test for categorical variables and the Student's t-test for continuous variables. The normality of the variables was confirmed. The enrollment date was established as the baseline point for each subject from April 2016 to March 2019. The follow-up period extended from April 2017 to March 2023. A Cox proportional hazards model was used to examine the association between WC, WC- related indices, BMI and the progression of kidney damage. BMI above the cutoff was included in the predictive model to determine whether it is a contributing factor in the relationship between WC and kidney disease progression. All statistical analyses were conducted using SAS version 9.4. A significance level of 0.05 and a 95% confidence interval (95% CI) were set for this study.

### Ethical consideration

A written consent form was collected from each participant during their visit. Participants were anonymized, and the sheet containing identifying information is stored and managed by the principal investigator of the research project. This study was approved by the research ethics committee of the Faculty of Medicine at the University of Tsukuba (approval number: 1000). It was conducted by following the Declaration of Helsinki.

## Results

**Fig 1:**
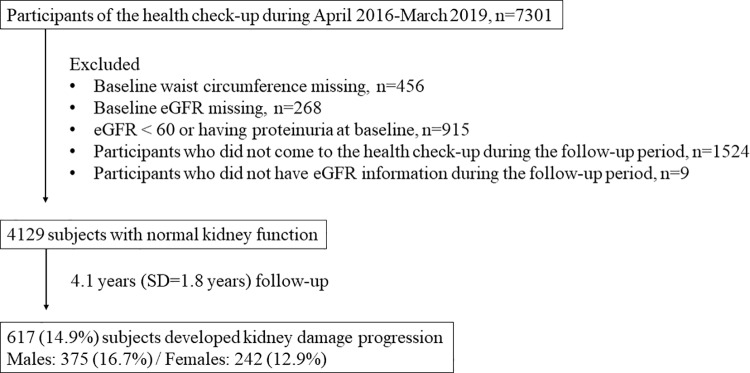
Study flow chart.

Fig 1 illustrates the study flowchart. Between April 2016 and March 2019, 7,301 participants attended the health checkup. After excluding individuals for missing baseline waist circumference (n=456), missing baseline eGFR (n=268), eGFR < 60 mL/min/1.73m^2^ or proteinuria at baseline (n=915), absence from health checkups during the follow-up period (n=1524), and missing eGFR information during the follow-up (n=9), 4,129 subjects with normal kidney function were enrolled in this study. During an average follow-up of 4.1 years (SD=1.8 years), 617 (14.9%) subjects developed kidney damage progression.

Table 1 presents the baseline characteristics of the subjects, further stratified by sex. The average age of the participants was 48.5 years, primarily placing them within the middle-aged category (40–64 years old). Of the total, 2,246 (54.4%) were males. Females were significantly older than males (50.2 years for females vs. 47.0 years for males, p<0.001). The average baseline eGFR was 79.7mL/min/1.73m^2^, indicative of a mildly decreased level of chronic kidney disease. BMI was significantly higher in males than in females (24.3 for males vs. 22.6 for females, p<0.001). The average baseline WC was 83.0 cm, with males showing significantly higher measurements than females (85.6 cm for males vs. 79.9 cm for females, p<0.001). According to JASSO and WHO definitions, the WC groups showed distinct distributions. Under the JASSO definition, 50.8% of males were categorized in the high WC group, significantly higher compared to 15.5% in females (p<0.001). Meanwhile, using the WHO definition, a higher proportion of females had a high WC, accounting for around 48.5%, in contrast to 30.0% for males (p<0.001). As for the WC-related indices, WHtR, BRI, conicity index, ABSI, and WWI were all significantly higher in females (WHtR: 0.51 for females vs. 0.50 for males, p<0.001; BRI: 0.67 for females vs. 3.50 for males, p<0.001; conicity index: 1.23 for females vs. 1.22 for males, p<0.001; ABSI: 0.080 for females vs. 0.078 for males, p<0.001; WWI: 10.8 for females vs. 10.2 for males, p<0.001;). Regarding comorbidities, males had higher proportions of hypertension, diabetes, and dyslipidemia (hypertension: 32.5% in males vs. 24.0% in females, p<0.001; diabetes: 10.3% in males vs. 4.7% in females, p<0.001; dyslipidemia: 44.0% in males vs. 31.8% in females, p<0.001). Males also reported higher levels of regular exercise but smoked more and drank more than females (regular exercise: 24.3% in males vs. 16.6% in females, p<0.001; smoking: 33.7% in males vs. 10.3% in females, p<0.001; alcohol: 64.1% in males vs. 33.9% in females, p<0.001).

**Table 1:** Baseline characteristics of overall and stratified subjects by sex.

	total, n=4129	males, n=2246	females, n=1883	p-value
Age, years	48.5 ± 14.2	47.0 ± 14.3	50.2 ± 13.8	<0.001
Age group, years				<0.001
<40	1145 (25.7%)	719 (32.0%)	426 (22.6%)	
40-64	2431 (58.9%)	1264 (56.3%)	1167 (62.0%)	
≧65	553 (13.4%)	263 (11.7%)	290 (15.4%)	
eGFR, mL/min/1.73m^2^	79.7 ± 13.2	79.7 ± 12.7	79.7 ± 13.8	0.962
BMI, kg/m^2^	23.6 ± 3.8	24.3 ± 3.7	22.6 ± 3.7	<0.001
BMI categories				<0.001
BMI<25 kg/m^2^	2859 (69.2%)	1402 (62.4%)	1457 (77.4%)	
BMI≧25 kg/m^2^	1270 (30.8%)	844 (37.6%)	426 (22.6%)	
Waist circumference, cm	83.0 ± 10.3	85.6 ± 9.7	79.9 ± 10.1	<0.001
Waist circumference group (JASSO)				<0.001
high WC	1432 (34.7%)	1140 (50.8%)	292 (15.5%)	
low WC	2697 (65.3%)	1106 (49.2%)	1591 (84.5%)	
Waist circumference group (WHO)				<0.001
high WC	1588 (38.5%)	674 (30.0%)	914 (48.5%)	
low WC	2541 (61.5%)	1572 (70.0%)	969 (51.5%)	
Waist-to-height ratio	0.51 ± 0.06	0.50 ± 0.06	0.51 ± 0.07	<0.001
Waist-BMI ratio	3.55 ± 0.26	3.54 ± 0.23	3.56 ± 0.29	0.009
Body roundness index	3.57 ± 1.25	3.50 ± 1.13	3.67 ± 1.37	<0.001
Conicity index	1.23 ± 0.08	1.22 ± 0.06	1.23 ± 0.09	<0.001
A body shape index	0.079 ± 0.004	0.078 ± 0.004	0.080 ± 0.005	<0.001
Weight-adjusted waist index	10.47 ± 0.75	10.2 ± 0.59	10.8 ± 0.81	<0.001
Hypertension, n(%)^a^	1181 (28.6%)	729 (32.5%)	452 (24.0%)	<0.001
Dyslipidemia, n(%)	1587 (38.4%)	989 (44.0%)	598 (31.8%)	<0.001
Diabetes, n(%)	321 (7.8%)	232 (10.3%)	89 (4.7%)	<0.001
Exercise, n(%)^b^	849 (20.8%)	539 (24.3%)	310 (16.6%)	<0.001
Smoking, n(%)^c^	939 (23.0%)	747 (33.7%)	192 (10.3%)	<0.001
Alcohol, n(%)^d^	2077 (50.3%)	1439 (64.1%)	638 (33.9%)	<0.001

^a^missing value, n=1;

^b^missing value, n=47;

^c^missing value, n=43;

^d^missing value, n=56

During the follow-up period, 617 (14.9%) participants experienced progression of kidney damage. The characteristics of participants with and without kidney damage progression are shown in Table 2. The average age was significantly higher in the progression group (51.46 ± 13.95 years) compared to the no-progression group (47.92 ± 14.14 years, p<0.001). Males were more prevalent in the progression group (60.8%) compared to the no-progression group (53.3%, p<0.001). BMI was higher in the progression group (24.3 ± 3.9 kg/m²) than in the no-progression group (23.5 ± 3.8 kg/m², p<0.001). Higher WC-related indicators including the JASSO and WHO classifications, WHtR and the WHtR classification were all indicated higher prevalence in the progression group. Other indices such as WC/BMI ratio, body roundness index, conicity index, and A body shape index showed minimal differences between the groups, with no statistically significant differences for conicity index, A body shape index, and weight-adjusted waist index. Regarding comorbidities, hypertension (38.2% vs. 26.9%, p<0.001), dyslipidemia (42.8% vs. 37.7%, p=0.018), and diabetes (10.2% vs. 7.3%, p=0.018) were significantly more prevalent in the progression group. There was no significant difference in exercise habits (p=0.235). Smoking and alcohol consumption were slightly more common in the progression group (76.8% for smoking and 51.7 for alcohol) compared to the no-progression group (76.1%, p=0.950 for smoking and 50.1%, p=0.574 for alcohol), though neither reached statistical significance.

**Table 2:** Baseline characteristics between participants with and without kidney damage progression.

	Progression of kidney damage, n=617	No progression of kidney damage, n=3512	p-value
Age, years	51.46 ± 13.95	47.92 ± 14.14	<0.001
Males, n%	375 (60.8%)	1871 (53.3%)	<0.001
Age group, years			<0.001
<40	123 (19.9%)	1022 (29.1%)	
40-64	390 (63.2%)	2041 (58.1%)	
≧65	104 (16.9%)	449 (12.8%)	
eGFR, mL/min/1.73m^2^	72.2 ± 13.9	81.0 ± 12.6	<0.001
BMI, kg/m^2^	24.3 ± 3.9	23.4 ± 3.8	<0.001
BMI categories			<0.001
BMI<25 kg/m^2^	362 (58.7%)	2497 (71.1%)	
BMI≧25 kg/m^2^	255 (41.3%)	1015 (28.9%)	
Waist circumference, cm	84.6 ± 10.4	82.7 ± 10.3	<0.001
Waist circumference group (JASSO)			<0.001
high WC	266 (43.1%)	1166 (33.2%)	
low WC	351 (56.9%)	2346 (66.8%)	
Waist circumference group (WHO)			0.004
high WC	270 (43.8%)	1318 (37.5%)	
low WC	347 (56.2%)	2194 (62.5%)	
Waist-to-height ratio	0.52 ± 0.06	0.51 ± 0.06	<0.001
Waist-to-height categories			0.041
high WHtR	315 (51.1%)	1633 (46.5%)	
low WHtR	302 (48.9%)	1879 (53.5%)	
WC/BMI ratio	3.51 ± 0.26	3.56 ± 0.26	<0.001
body roundness index	3.74 ± 1.25	3.54 ± 1.24	<0.001
Conicity index	1.23 ± 0.07	1.23 ± 0.08	0.102
A body shape index	0.079 ± 0.004	0.079 ± 0.004	0.561
Weight-adjusted waist index	10.49 ± 0.76	10.46 ± 0.75	0.362
Hypertension, n(%) a	236 (38.2%)	945 (26.9%)	<0.001
Dyslipidemia, n(%)	264 (42.8%)	1323 (37.7%)	0.018
Diabetes, n(%)	63 (10.2%)	258 (7.3%)	0.018
Exercise, n(%) b	474 (76.8%)	2759 (78.6%)	0.235
Smoking, n(%) c	474 (76.8%)	2673 (76.1%)	0.950
Alcohol, n(%) d	319 (51.7%)	1758 (50.1%)	0.574

Table 3 displays various indicators of WC and the hazard risk for the progression of kidney damage, stratified by sex. After adjusting for age, the body roundness ratio indicated a significant risk for the progression of kidney damage among males (hazard ratio, HR = 1.13, p = 0.010). After adjusting for age, sex, comorbidities, and health-related lifestyles, both WC and its categorical definitions from JASSO and WHO showed significant risks for the progression of kidney damage among males (hazard ratio, HR = 1.01, p-value = 0.045 for continuous WC; HR = 1.26, p-value = 0.035 for high WC as defined by the JASSO definition; HR = 1.39, p-value = 0.005 for high WC as defined by the WHO definition). In contrast, a higher Waist-BMI ratio and ABSI demonstrated a protective effect against the progression of kidney damage among males (HR = 0.37, p-value < 0.005 for higher Waist-BMI ratio; HR = 0.67, p-value = 0.019 for higher ABSI group). No significant association was observed between WC and the progression of kidney damage among females. The association between WC and kidney damage progression disappeared after including the BMI above the cutoff in the predictive models (S1-S3 Tables in S1 File).

**Table 3:** Cox Proportional Hazard Model for waist circumference indicators and progression of kidney damage, stratified by sex.

	Model 1^a^			Model 2^b^			Model 3^c^		
	aHR	95%CI	p-value	aHR	95%CI	p-value	aHR	95%CI	p-value
**Males**									
Waist circumference	1.02	1.00-1.03	**0.007**	1.01	1.0-1.02	**0.043**	1.01	1.00-1.02	**0.044**
JASSO definition									
high WC (ref = low WC)	1.33	1.08-1.63	**0.007**	1.27	1.02-1.58	**0.031**	1.27	1.02-1.57	**0.034**
WHO definition									
high WC (ref = low WC)	1.44	1.16-1.77	**0.001**	1.39	1.11-1.74	**0.004**	1.39	1.11-1.74	**0.005**
WHtR≥0.52 (ref=WHtR<0.52)	1.23	0.99-1.52	**0.050**	1.17	0.94-1.45	0.166	1.16	0.93-1.45	0.175
WC/BMI ratio	0.34	0.22-0.54	**<0.001**	0.38	0.24-0.61	**<0.001**	0.38	0.24-0.61	**<0.001**
Body roundness index	1.13	1.03-1.23	**0.010**	1.10	0.99-1.21	0.060	1.10	0.99-1.21	0.069
Conicity index≥1.275 (ref=conicity index<1.275)	0.95	0.74-1.22	0.691	0.91	0.70-1.17	0.451	0.92	0.71-1.19	0.525
ABSI≥0.0822 (ref=ABSI<0.0822)	0.67	0.48-0.94	**0.019**	0.67	0.48-0.94	**0.020**	0.68	0.49-0.95	**0.025**
Weight-adjusted waist index	0.96	0.79-1.17	0.683	0.90	0.73-1.11	0.316	0.89	0.73-1.10	0.275
**Females**									
Waist circumference	1.01	0.99-1.02	0.130	1.01	0.99-1.02	0.265	1.01	0.99-1.02	0.352
JASSO definition									
high WC (ref = low WC)	1.32	0.96-1.81	0.088	1.27	0.91-1.75	0.157	1.25	0.90-1.73	0.191
WHO definition									
high WC (ref = low WC)	1.18	0.91-1.53	0.206	1.14	0.88-1.48	0.334	1.11	0.85-1.45	0.424
WHtR≥0.49 (ref=WHtR<0.49)	1.03	0.79-1.36	0.811	0.99	0.75-1.31	0.943	0.97	0.74-1.29	0.859
WC/BMI ratio	0.63	0.40-0.99	**0.045**	0.67	0.42-1.05	0.082	0.68	0.43-1.07	0.094
Body roundness index	1.07	0.97-1.17	0.163	1.05	0.95-1.16	0.337	1.04	0.94-1.15	0.417
Conicity index≥1.285 (ref=conicity index<1.285)	0.92	0.68-1.22	0.551	0.88	0.66-1.19	0.410	0.89	0.66-1.19	0.416
ABSI≥0.0795 (ref=ABSI<0.0795)	0.89	0.68-1.16	0.405	0.89	0.68-1.16	0.385	0.89	0.68-1.16	0.378
weight-adjusted waist index	1.00	0.84-1.20	0.960	0.98	0.82-1.17	0.820	0.97	0.81-1.16	0.754

^a^Model 1: adjusted for age and sex

^b^Model 2: adjusted for model 1 + diabetes, hypertension, and dyslipidemia

^c^Model 3: adjusted for model 2 + exercise, smoking, and alcohol consumption

Table 4 further illustrates the risk of BMI and the progression of kidney damage, stratified by sex. After adjusting for age, comorbidities, and lifestyle factors, the higher BMI group, defined as a BMI ≥ 25 kg/m^2^ for males and BMI ≥ 23 kg/m^2^ for females, was associated with a significantly increased risk of kidney damage progression among males but not in females (HR=1.69, p-value<0.001 for males and HR=1.25, p-value=0.103 for females).

**Table 4:** Cox Proportional Hazard Model for the BMI and the risk of progression of kidney damage stratified by sex.

	Model 1^a^			Model 2^b^			Model 3^c^		
	aHR	95%CI	p-value	aHR	95%CI	p-value	aHR	95%CI	p-value
Males									
BMI≥25 kg/m^2^	1.73	1.41-2.12	**<.0001**	1.69	1.37-2.10	**<.0001**	1.69	1.36-2.09	**<.0001**
Females									
BMI≥23 kg/m^2^	1.31	1.01-1.69	**0.04**	1.26	0.97-1.64	0.081	1.25	0.96-1.62	0.103

^a^Model 1: adjusted for age and sex

^b^Model 2: adjusted for model 1 + diabetes, hypertension, and dyslipidemia

^c^Model 3: adjusted for model 2 + exercise, smoking, and alcohol consumption

## Discussion

After four years of follow-up, this study identified an association between increased WC and the risk of kidney damage progression among males, regardless of the WC risk category definition used. This association was not observed in females. However, WC-related indices showed no significant associations. After incorporating BMI into the analysis, the association between WC and kidney damage progression disappeared. This finding highlights the independent effect of BMI on kidney damage progression among the study subjects.

WC is an indicator of abdominal obesity and shows a correlation with visceral adipose tissue, which is linked to the development of metabolic complications [30–32]. In Japan, several studies have examined the association between WC and CKD. However, their conclusions have been inconsistent due to variations in the WC cutoff points. Yamasaki et al. conducted a cohort study and found that a WC above the fourth quartile (Q4), defined as 89.6 cm, was associated with a 1.66-fold increased risk of CKD in middle-aged males, but no significant association was found in females [33]. Kuma et al. found that a WC exceeding 80 cm was associated with a significantly higher risk of CKD in middle-aged males in Japan [34]. In this study, the WC cutoff points officially published by WHO and JASSO were utilized. Both were associated with a significant risk of CKD progression in males, with hazard ratios ranging from 1.27 to 1.39, but no significant risk was observed in females. These findings align with previous studies and confirm the predictive value of the published cutoff points by WHO and JASSO. JASSO's definition sets a lower cutoff point for WC in males compared to the WHO definition. A previous study that compared different WC cutoff points found that a cutoff of more than 85 cm was more effective in detecting the incidence of diabetes than the JASSO definition [35]. In this study, compared to the JASSO definition, the WHO definition revealed a higher hazard ratio for the association between high WC and the progression of kidney damage in males. This suggests that using a more stringent definition can improve the detection of risk differences.

The cutoff points for WHtR were set at 0.52 for males and 0.49 for females in this study. However, no significant association was found between WHtR and CKD. A previous study showed that a WHtR above Q4 (greater than 0.52) was linked to a 1.61-fold increased risk of CKD in Japanese males, while a WHtR above Q4 (greater than 0.55) was associated with a 2.38-fold increased risk in Japanese females [33]. The key difference in our study is that it focused on the working population, which represents a generally healthier group. Although no clear association was found between WHtR and CKD in our study, a trend toward significance was observed in males, suggesting a borderline association.

Waist-BMI ratio and ABSI exhibited a reverse effect on CKD. The Waist-BMI ratio, which divides WC by BMI, has not been studied in relation to CKD risk. However, one previous study found a J-shaped relationship between the Waist-BMI ratio and mortality, suggesting that this indicator may be more effective for identifying higher-risk obesity phenotypes [10]. Given that the population in this study tends to be healthy, further research is needed to examine the predictive value of the Waist-BMI ratio for CKD. ABSI is an indicator that considers WC, BMI, and height, with higher ABSI indicating greater abdominal adiposity. However, a higher ABSI may also reflect a lower BMI, potentially offering protection against CKD risk. Previous studies have shown that higher ABSI can predict CKD risk in older adults [36], but it may not be an ideal predictor for CKD in healthy middle-aged populations. A recent cross-sectional study investigated the association between BRI and CKD, reporting a moderate association with an odds ratio of approximately 1.3 [37]. Our longitudinal study found that BRI did not reach statistical significance, although it approached significance, indicating that BRI may not be a reliable predictor of CKD progression.

Compared to the hazard ratios of WC and WC-related indices, BMI emerged as the strongest predictor for CKD. The association between WC and the risk of kidney damage progression disappeared after incorporating BMI into the analysis, underscoring the strong correlation between BMI and WC in assessing kidney damage risk. Although BMI and WC are strongly correlated, they serve distinct purposes as indicators [38]. BMI measures overall body adiposity based on height and weight, whereas WC specifically targets abdominal adiposity. These indicators are linked through common pathways in the progression of kidney damage. Insulin resistance and inflammation can increase oxidative stress, potentially causing cellular damage and impairing kidney structures [39]. The accumulation of visceral fat may activate the renin-angiotensin system, leading to adipose deposition, enhanced ultrafiltration, and increased sodium reabsorption in the kidneys [40]. In this study, both BMI and WC were found to significantly increase the risk of kidney damage progression, with BMI having a greater impact on predictability.

No significant relationship was found between WC and kidney damage in females, mirroring findings from a previous Japanese study [38]. In this study, baseline characteristics indicate that females generally had fewer comorbidities and lower BMI than males, suggesting a healthier condition. It has also been reported that females experience a slower progression of kidney damage than males, potentially due to estrogen protection during premenopausal status [41]. The lower incidence of kidney damage in females may contribute to reduced statistical power in detecting significant effects. Further studies with longer follow-up periods should be conducted to explore the impact of WC on kidney damage among females with lower WC.

This study boasts several strengths and limitations. The first strength lies in the demographics of the study participants, who were recruited from annual health checkups, representing a relatively healthier population that is pertinent from an early prevention perspective. The second strength is the consistent return rate of approximately 70% among participants in subsequent annual health checkups. Thirdly, the large sample size enhances the reliability of the causal inferences drawn. As for the limitations, the first is the limited number of subjects within the normal BMI range but with a large WC, which may contribute to the failure to observe significant risks for kidney damage. Secondly, the statistical power to detect the progression of kidney damage in females may be insufficient. Third, given that the study population had normal kidney function, the follow-up period may not have been adequate. Lastly, information about fatty liver, which is found to be associated with an increased risk of CKD progression, was not collected and adjusted in this study. Overall, future studies would benefit from a longer follow-up period to assess these factors more effectively.

## Conclusions

In conclusion, this study has demonstrated that a large waist circumference significantly contributes to the progression of kidney damage among males. Nevertheless, it also highlights BMI as a reliable predictor of kidney damage. The author recommends maintaining normal ranges for both BMI and waist circumference to mitigate the risk of kidney damage progression.

## Supporting information

S1 File S1 Table. Cox Proportional Hazard Model for the continuous waist circumference and the risk of progression of kidney damage stratified by BMI. S2 Table. Cox Proportional Hazard Model for the categorical waist circumference (JASSO definition) and the risk of progression of kidney damage stratified by BMI. S3 Table. Cox Proportional Hazard Model for the categorical waist circumference (WHO definition) and the risk of progression of kidney damage stratified by BMI. (DOCX)

S1 Data Analyzed data used in this study. (CSV)
