# Supplementary material for: A prospective study of comparing waist circumference and BMI as predictors for the kidney damage progression
Source: PLoS One. 2025 Apr 29;20(4):e0321012. doi: 10.1371/journal.pone.0321012 (PMC12040278; doi:10.1371/journal.pone.0321012)
Supplement: S1 File — (DOCX) [file pone.0321012.s001.docx]

S1 Table. Cox Proportional Hazard Model for the continuous waist circumference and the risk of progression of kidney damage stratified by BMI

|  | Model 1 ^a^ | | | | Model 2 ^b^ | | | Model 3 ^c^ | | |
| --- | --- | --- | --- | --- | --- | --- | --- | --- | --- | --- |
|  | **n** | aHR | 95%CI | p-value | aHR | 95%CI | p-value | aHR | 95%CI | p-value |
| **Males** |  |  |  |  |  |  |  |  |  |  |
| BMI<25 |  |  |  |  |  |  |  |  |  |  |
| waist circumference | 1402 | 0.99 | 0.96-1.01 | 0.235 | 0.99 | 0.96-1.01 | 0.213 | 0.98 | 0.96-1.01 | 0.193 |
| BMI≧25 |  |  |  |  |  |  |  |  |  |  |
| waist circumference | 844 | 0.993 | 0.97-1.01 | 0.481 | 0.993 | 0.97-1.01 | 0.533 | 0.994 | 0.97-1.02 | 0.563 |
| **Females** |  |  |  |  |  |  |  |  |  |  |
| BMI<25 |  |  |  |  |  |  |  |  |  |  |
| waist circumference | 1457 | 0.99 | 0.97-1.01 | 0.378 | 0.99 | 0.97-1.01 | 0.311 | 0.99 | 0.97-1.01 | 0.224 |
| BMI≧25 |  |  |  |  |  |  |  |  |  |  |
| waist circumference | 426 | 1.00 | 0.97-1.03 | 0.999 | 1.00 | 0.97-1.03 | 0.985 | 1.00 | 0.97-1.03 | 0.992 |
| ^a^ Model 1: adjusted for age and sex | | | | | | | | | | |
| ^b^ Model 2: adjusted for modle 1 + diabetes, hypertension, and dyslipidemia | | | | | | | | | | |
| ^c^ Model 3: adjusted for model 2 + exercise, smoking | | | | | | | | | | |

S2 Table. Cox Proportional Hazard Model for the categorical waist circumference (JASSO definition) and the risk of progression of kidney damage stratified by BMI

|  | Model 1 ^a^ | | | Model 2 ^b^ | | | Model 3 ^c^ | | |
| --- | --- | --- | --- | --- | --- | --- | --- | --- | --- |
|  | aHR | 95%CI | p-value | aHR | 95%CI | p-value | aHR | 95%CI | p-value |
| **Males** |  |  |  |  |  |  |  |  |  |
| BMI<25 |  |  |  |  |  |  |  |  |  |
| high WC (ref = low WC) | 0.80 | 0.57-1.13 | 0.203 | 0.78 | 0.56-1.11 | 0.167 | 0.78 | 0.55-1.10 | 0.158 |
| BMI≧25 |  |  |  |  |  |  |  |  |  |
| high WC (ref = low WC) | 1.17 | 0.63-2.15 | 0.620 | 1.22 | 0.66-2.25 | 0.534 | 1.22 | 0.66-2.26 | 0.530 |
| **Females** |  |  |  |  |  |  |  |  |  |
| BMI<25 |  |  |  |  |  |  |  |  |  |
| high WC (ref = low WC) | 0.95 | 0.44-2.06 | 0.904 | 0.95 | 0.44-2.06 | 0.899 | 0.82 | 0.36-1.88 | 0.641 |
| BMI≧25 |  |  |  |  |  |  |  |  |  |
| high WC (ref = low WC) | 1.00 | 0.63-1.61 | 0.990 | 1.02 | 0.63-1.63 | 0.950 | 1.06 | 0.66-1.72 | 0.808 |
| ^a^ Model 1: adjusted for age and sex | | | | | | | | | |
| ^b^ Model 2: adjusted for modle 1 + diabetes, hypertension, and dyslipidemia | | | | | | | | | |
| ^c^ Model 3: adjusted for model 2 + exercise, smoking | | | | | | | | | |

S3 Table. Cox Proportional Hazard Model for the categorical waist circumference (WHO definition) and the risk of progression of kidney damage stratified by BMI

|  | Model 1 ^a^ | | | Model 2 ^b^ | | | Model 3 ^c^ | | |
| --- | --- | --- | --- | --- | --- | --- | --- | --- | --- |
|  | aHR | 95%CI | p-value | aHR | 95%CI | p-value | aHR | 95%CI | p-value |
| **Males** |  |  |  |  |  |  |  |  |  |
| BMI<25 |  |  |  |  |  |  |  |  |  |
| high WC (ref = low WC) | 1.00 | 0.54-1.85 | 0.989 | 1.00 | 0.54-1.86 | 1.000 | 1.012 | 0.55-1.88 | 0.969 |
| BMI≧25 |  |  |  |  |  |  |  |  |  |
| high WC (ref = low WC) | 0.95 | 0.70-1.30 | 0.758 | 0.95 | 0.69-1.31 | 0.747 | 0.94 | 0.68-1.30 | 0.690 |
| **Females** |  |  |  |  |  |  |  |  |  |
| BMI<25 |  |  |  |  |  |  |  |  |  |
| high WC (ref = low WC) | 0.90 | 0.65-1.24 | 0.525 | 0.882 | 0.64-1.22 | 0.445 | 0.85 | 0.62-1.18 | 0.344 |
| BMI≧25 |  |  |  |  |  |  |  |  |  |
| high WC (ref = low WC) | 1.99 | 0.27-14.52 | 0.499 | 1.95 | 0.27-14.27 | 0.511 | 2.13 | 0.29-15.65 | 0.457 |
| ^a^ Model 1: adjusted for age and sex | | | | | | | | | |
| ^b^ Model 2: adjusted for modle 1 + diabetes, hypertension, and dyslipidemia | | | | | | | | | |
| ^c^ Model 3: adjusted for model 2 + exercise, smoking | | | | | | | | | |
